# Supplementary material for: Behaviourally-Informed Two-Way Text Messaging to Improve Return to HIV Care in South Africa: Evidence from a Randomised Controlled Trial
Source: AIDS Behav. 2025 Jul 11;29(11):3661–72. doi: 10.1007/s10461-025-04808-6 (PMC12500771; doi:10.1007/s10461-025-04808-6)
Supplement: Supplementary file 1 — Supplementary Material 1 [file 10461_2025_4808_MOESM1_ESM.docx]

**Supplementary Table 1** Association between text message delivery and participant characteristics

| **Participant characteristics** | **Text message delivery- Yes** | | **Text message delivery- No** | | **Chi-squared test** | **P-value** |
| --- | --- | --- | --- | --- | --- | --- |
| **Age at randomization (years)** | **N=2170** | **Percent (%)** | **N=1521** | **Percent (%)** |  |  |
| 18-24 | 157 | 54.33 | 132 | 45.67 | 3.3507 | 0.187 |
| 25-49 | 1594 | 58.80 | 1117 | 41.20 |  |  |
| ≥50 | 419 | 60.64 | 272 | 39.36 |  |  |
| **Gender** |  |  |  |  |  |  |
| Female | 1408 | 58.67 | 992 | 41.33 | 0.0169 | 0.897 |
| Male | 762 | 59.02 | 529 | 40.98 |  |  |
| **ART duration (months)** |  |  |  |  |  |  |
| <6 | 350 | 65.06 | 188 | 34.94 | 12.6014 | 0.002*** |
| 6-12 | 203 | 61.70 | 126 | 38.30 |  |  |
| >12 | 1617 | 57.26 | 1207 | 42.74 |  |  |
| **Treatment interruption stratification (months)** |  |  |  |  |  |  |
| <3 | 1269 | 59.77 | 854 | 40.23 | 1.9902 | 0.158 |
| ≥3 | 901 | 57.46 | 667 | 42.54 |  |  |
| **Enrollment in DMOC^a^** |  |  |  |  |  |  |
| Yes | 785 | 57.93 | 570 | 42.07 | 0.6507 | 0.420 |
| No | 1385 | 59.29 | 951 | 40.71 |  |  |
| **Priority clinic** |  |  |  |  |  |  |
| No | 978 | 59.63 | 662 | 40.37 | 0.8647 | 0.352 |
| Yes | 1192 | 58.12 | 859 | 41.88 |  |  |
| **Sub-district** |  |  |  |  |  |  |
| Blouberg | 269 | 56.87 | 204 | 43.13 | 1.5246 | 0.677 |
| Lepelle-Nkumpi | 307 | 58.70 | 216 | 41.30 |  |  |
| Molemole | 226 | 61.08 | 144 | 38.92 |  |  |
| Polokwane | 1368 | 58.84 | 957 | 41.16 |  |  |

***p<0.01

^a^ DMOC - Differentiated models of care
